# Extraordinary Case of Pregnancy in Which the Fœtus Was Discharged through the Abdominal Viscera

**Published:** 1830

**Authors:** Joseph A. Toy

**Affiliations:** Virginia


6. *Extraordinary case of Pregnancy in which the Fœtus was discharged through the abdominal parietes. By* Joseph A. Toy, M. D. *of Virginia.*

On the morning of the 1st November, 1828, Dr. T. was requested to see a lady in the county of Warwick, who was represented to have been in labor three days; the waters having been discharged on the evening before. She was found suffering extremely with a dull heavy pain across the abdomen. The abdomen was swelled, tense and hot—pulse small and quick—great restlessness and anxiety—os tincœ soft and somewhat dilated—the external parts swelled and inflamed. Emollient fomentations were applied, and the secale cor. was given, one-tenth of a grain every twenty minutes, until 2 drams were administered, a sudorific anodyne being conjoined with the first dose. No effect was produced by the sec. cor. The pain subsided, and the febrile symptoms were relieved by the fomentations. No fœtus could be discovered per vaginam, but a tumour resembling a child's head could be distinctly felt, in the abdomen near the navel. Her health suffered severely until the 12th, when a very offensive discharge from the vagina appeared.

Dr. T. saw her again in January, 1829. No physician had seen her for several weeks; the discharge continued, and within the last three weeks, several small bones were discharged, which were found to be an entire fore arm and hand, and the metacarpal bones and fingers of the opposite arms. She was feeble, cachectic, and edæmatous. These symptoms were very much relieved by a tonic course of treatment.

On the 7th May, Dr, T. saw her again. Six or seven weeks before, a small discoloured spot had appeared near the navel, and she suffered considerable pain in this part. In this place a slight aperture existed, into which a fine probe could be introduced and from which a small discharge issued. The discharge per vaginam had ceased. On the 6th of July, the opening being closed, an incision was made and the probe when introduced came in contact with bone. Her health now gave way rapidly—hectic fever with irregular exacerbations every day, uneasiness over the whole abdomen, great emaciation and debility. On the 12th of August she reluctantly submitted to an operation, which had been repeatedly urged for several months, as the only mode of relieving her. Dr. T. assisted by Banks, performed the operation.

The aperture had increased in size considerably, being about two lines in diameter at the surface. I made the first incision about four inches long, in the direction of the linea alba, commencing two inches above the umbilicus, and one inch to the right of it. This was continued through the parietes of the abdomen and uterus into the cavity of the latter, the incision in the uterus being more than two inches in length. This incision I crossed in its centre, by another at right angles with it of equal length and depth. The top of the cranium was found presenting, the bones appearing to be very firmly united.

I attempted to introduce a pair of small obstetrical forceps, with the view of removing the head at once. This, however, was found to be impracticable without using more force than I judged prudent, as the uterus was firmly and rigidly contracted around it. I used therefore, a pair of strong forceps from a pocket case, and with these succeeded in detaching and removing the bones of the cranium separately and successively ; all the other bones of the fœtus remaining. We found that adhesive inflammation had united the uterus to the parietes of the abdomen for some distance around the opening, the diameter of the circle of adhesion was more than an inch. The operation occupied about fifteen minutes. After repeatedly washing the part, the edges of the wound were brought together, and dressed with adhesive plaster. Stitches were found unnecessary. Over this, pledgets of lint and a compress of old linen were applied, and a roller passed round the abdome n. Before the wound was dressed the lady expressed herself entirely relieved from the uneasiness which the presence of the bones had so long occasioned. She complained of some pain about the uterus.

On our visit the the next day, she was in all respects doing well. The pain which she complained of the preceding day had continued for some hours, but she was now entirely easy, and expressed in strong terms the relief which she had felt since the removal of the bones.

In six weeks she was entirely well, and had visited several of her neighbours. The opening which had existed previous to the operation was not entirely filled up, but the incisions united by the first intention.

I saw this lady in December last—she was then in fine health and very fleshy. The catamenial discharge had returned and was now regular.

This case could not have been one of extra-uterine conception, for the early history of the case together with the discharge of the soft parts and bones per vaginam, prove incontestibly that it was uterine, even if the operation had not demonstrated the fact. Neither can it be believed that there had been rupture of the uterus at any period during the progress of this case, for when the bones were removed they were firmly enclosed in the uterus, and there was no other opening but the passage externally, for which we think there is little difficulty in accounting. The pressure of the bones against the walls of the uterus, produced inflammation and snppuration, which, passing by continuity of surface to the parietes of the abdomen occasioned the aperture. The inflammation thus exeited also produced adhesion between the uterus and abdominal parietes, which union must still exist—indeed, when I last examined her, the parietes of the abdomen were considerbly drawn in by this attachment.

*American Journal of the Med. Sci.*

